# Bilateral Vallecular Cysts as a Cause of Dysphagia: Case Report and Literature Review

**DOI:** 10.1155/2010/697583

**Published:** 2010-12-12

**Authors:** Jonathan J. Romak, Steven M. Olsen, Cody A. Koch, Dale C. Ekbom

**Affiliations:** Department of Otorhinolaryngology, Head and Neck Surgery, Mayo Clinic, 200 First Street SW, Rochester, MN 55905, USA

## Abstract

Cysts of the vallecula are rare, accounting for 10.5% to 20.1% of all laryngeal cysts. Vallecular cysts may present with diverse symptoms affecting the voice, airway, and swallowing. We describe the evaluation and treatment of a 70-year-old woman who presented with dysphagia caused by large bilateral vallecular cysts.

## 1. Introduction

In infants and children, vallecular cysts present most commonly with stridor and feeding difficulty but may cause life-threatening airway obstruction [1, 2]. In adults, most vallecular cysts are asymptomatic but may present with globus, voice change, dysphagia, odynophagia, or dyspnea [3, 4]. Vallecular cysts may also be discovered during administration of anesthesia, where they may obscure the view of the glottis and cause difficult endotracheal intubation [5, 6]. We report here a case of bilateral vallecular cysts as a cause of dysphagia.

## 2. Case Report

**Figure 1:**
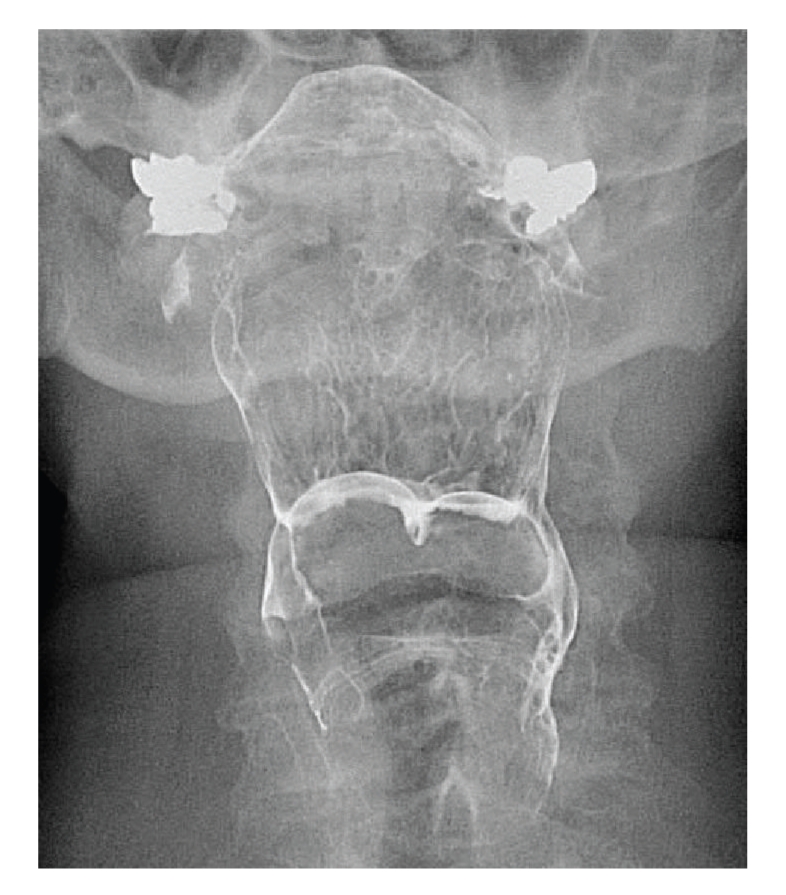
Coronal fluoroscopic image from a modified barium swallow study showing two masses in the vallecula coated in contrast material.

A 70-year-old woman was referred to our laryngology service after vallecular lesions were identified on a barium swallow study performed after several months of dysphagia (Figure 1). The patient reported dysphagia to solids and liquids with mild intermittent dysphonia. All other clinical findings were normal.

Interestingly, the patient also reported a history of angioedema, having presented to the emergency department 2 months earlier with tongue swelling. At that time, her enalapril was discontinued, and she was successfully treated with corticosteroids and antihistamines. This occurrence was followed by multiple episodes of lip and periorbital swelling, which ceased when she was started on daily cetirizine. Findings on general physical examination and vital signs were within normal limits. Laboratory evaluation was unremarkable except for an elevated total serum IgE level.

**Figure 2:**
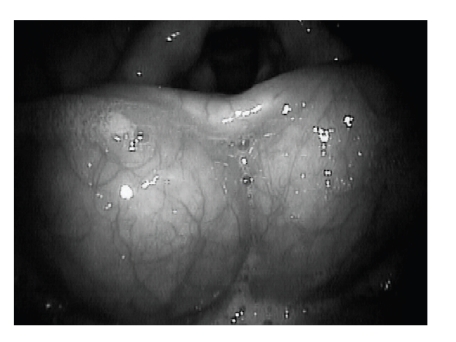
Direct laryngoscopic view of the vallecula showing large, bilateral cysts.

**Figure 3:**
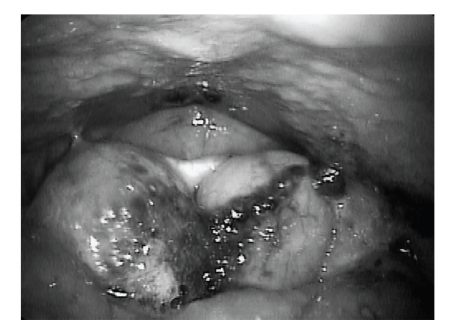
Preoperative flexible laryngoscopic view of functional endoscopic evaluation of swallowing.

On flexible laryngoscopy, 2 cysts were seen between the base of the tongue and the epiglottis, pushing the epiglottis posteriorly (Figure 2). Both cysts were approximately 2 cm in diameter, benign in appearance, nonerythematous, and with prominent overlying vasculature. Functional endoscopic evaluation of swallowing was performed (Figure 3). A portion of the swallowed food became lodged between the cysts and base of the tongue. With multiple swallows, the bolus was cleared. Water pooled between the cysts and base of tongue. A modified barium swallow study demonstrated retention of contrast in the vallecula. No laryngeal penetration or aspiration was noted.

**Figure 4:**
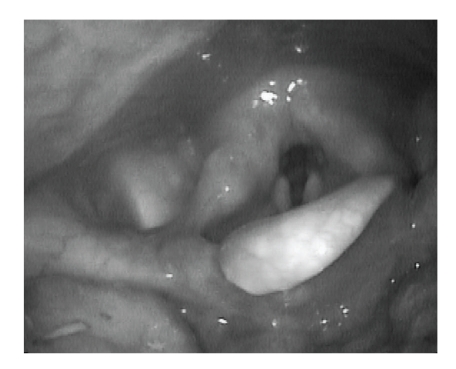
Six week postoperative flexible laryngoscopic view of the healed supraglottis.

Because of these findings, the patient underwent direct laryngoscopy and cyst excision. A Lindholm laryngoscope was used for exposure of the vallecula. The cysts were then removed in their entirety using a CO_2_ laser. The procedure was uncomplicated. At 6-week followup, the patient reported considerable improvement of her dysphagia. The vallecula was well healed with no evidence of cyst remnants (Figure 4). Findings on a postoperative barium swallow study were normal.

## 3. Discussion

Vallecular cysts, also called epiglottic mucus retention cysts or base of tongue cysts, arise when the duct of a mucous gland or lingual tonsillar crypt becomes obstructed and dilates [1, 2]. These cysts have therefore been classified as ductal cysts, retention cysts, and lymphoepithelial cysts and are caused by inflammation, irritation, or trauma [3, 4]. Ductal cysts may occur at any location lined by mucosa and can be found at any site in the larynx other than the free edge of the true vocal cords [3]. Ductal cysts are the most common laryngeal cysts and occur most frequently at the true vocal fold, followed by the epiglottis and vallecula [3, 4]. Vallecular cysts resembling tonsillar crypts due to associated lymphoid tissue have been separately classified as lymphoepithelial cysts and may also occur in the aryepiglottic fold, vestibule, and piriform sinus [3]. Given this pathogenesis, it would not be surprising for multiple cysts to codevelop. As stated by DeSanto et al. [4], “vallecular cysts are often multiple.” However, to our knowledge, no specific cases of multiple vallecular cysts have been reported in the literature.

Additionally, an association has recently been established between infected vallecular cysts and severe supraglottic infection including epiglottitis [1]. This patient's history of angioedema raises the question of whether there might be an association between that disease process and the formation of vallecular cysts.

Infants with vallecular cysts are considered to be at risk of airway obstruction and death [2]. Therefore, all such cysts in infants and children should be removed surgically, with marsupialization via CO_2_ laser or electrocautery being the most commonly used method [1, 2, 7]. It is our bias to remove the cyst in its entirety to avoid recurrence as a result of epithelial remnants. Other authors have shared this preference [3, 8] and have posited that use of a CO_2_ laser may be superior because of potential vaporization of the epithelial lining [8].

In adults, vallecular cysts are more common but less dangerous. The peak incidence is in the fifth decade of life, and the majority of cysts occur in men [2, 3]. Nearly two-thirds of vallecular cysts are asymptomatic and are diagnosed incidentally on routine laryngeal examination [3]. The incidence of vallecular cysts on laryngoscopy has been reported as 1 in 1,250 to 1 in 4,200, but the true incidence is difficult to estimate [5]. 

In the case described here, the patient's large, bilateral vallecular cysts caused dysphagia to both solids and liquids. Although rare, vallecular cysts should be considered in the workup of dysphagia. Additionally, vallecular cysts factor into the differential diagnoses of voice difficulty, odynophagia, and dyspnea. Managing vallecular cysts via direct laryngoscopy and excision may lead to resolution of symptoms.

## 4. Conclusion

Cysts of the vallecula may account for an array of clinical symptoms. In adults, vallecular cysts are most often asymptomatic and discovered on routine laryngoscopy or during induction of anesthesia. However, globus, dysphonia, dysphagia, odynophagia, and dyspnea may occur. In evaluating these symptoms, the presence of a vallecular cyst should be considered. If a vallecular cyst is found, complete transoral laser excision will often result in cyst resolution and improved symptoms.
